# Ectopic ERK expression induces phenotypic conversion of C10 cells and alters DNA methyltransferase expression

**DOI:** 10.1186/1756-0500-5-217

**Published:** 2012-05-04

**Authors:** Ryan L Sontag, Thomas J Weber

**Affiliations:** 1 Systems Toxicology, Pacific Northwest National Laboratory, 790 6th Street, J4-02, Richland, WA, 99354, USA

**Keywords:** Epigenetics, ERK, DNMT

## Abstract

**Background:**

Many lung carcinogens activate mitogen activated protein kinase (MAPK) pathways and DNA methyltransferases (DNMTs) are under investigation as therapeutic targets for lung cancer. Our goal is to determine whether C10 type II alveolar epithelial cells are a sensitive model to investigate ERK-dependent transformation and DNMT expression patterns in experimental lung cancer.

**Findings:**

Ectopic expression of an extracellular signal regulated kinase (ERK)-green fluorescent protein (ERK1-GFP) induces acquisition of growth in soft agar that is selectively associated with latent effects on the expression of DNA methyl transferases (DNMT1 and 3b), xeroderma pigmentosum complementation group A (XPA), DNA-dependent protein kinase catalytic subunit (DNA-PKcs), increased phosphatase activity and enhanced sensitivity to 5-azacytidine (5-azaC)-mediated toxicity, relative to controls.

**Conclusions:**

Ectopic expression of ERK alone is sufficient to promote phenotypic conversion of C10 cells associated with altered DNMT expression patterns and sensitivity to DNMT inhibitor. This model may have applications for predicting sensitivity to DNMT inhibitors.

## Findings

**Figure 1:**
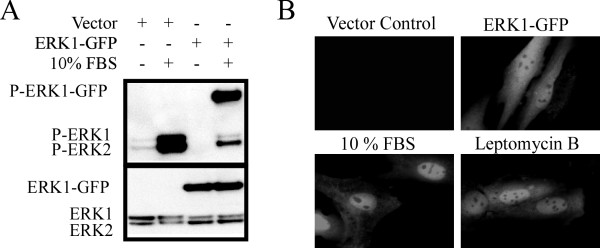
**Panel A: Expression of ERK1-GFP chimera was confirmed by Western blot analysis (bottom).** Phosphorylation of both ERK1-GFP and endogenous ERKs is increase by growth factor stimulation (FBS), relative to quiescent controls (top). **Panel B:** Epifluorescent microscopy confirmed ERK1-GFP nuclear translocation following stimulation with FBS, relative to unstimulated ERK1-GFP cells. Blocking nuclear export with leptomycin B (30 min treatment) resulted in retention of ERK1-GFP in the nucleus as expected. Similar results were observed in two independent experiments. Methods for retroviral expression of ERK1-GFP can be found in [15].

Many lung carcinogens activate the extracellular signal regulated kinase (ERK) [1] and in some model systems constitutive overexpression of ERK can induce transformation [2-6]. However, it is unclear whether ERK alone can modulate cell transformation responses in lung type II alveolar epithelial cells. In addition, cell transformation induced by ERK overexpression does not correlate with ERK activity [7], suggesting an important role for secondary regulatory events. Murine C10 type II alveolar epithelial cells have been used as an *in vitro* model to investigate molecular determinants of lung cell physiology and pathophysiology [8-11]. C10 type II alveolar epithelial cells are a non-tumorigenic cell line derived from normal BALB/c mouse lung tissue and do not contain native Ras mutations [12,13]. Type II features include the presence of lamellar bodies, the biosynthesis of surfactant, proliferation that is contact inhibited and anchorage-dependent growth [14]. Here we ectopically expressed an ERK1-GFP chimera in C10 cells using retroviral technology as previously described [15] and asked whether ectopic ERK expression induced phenotypic conversion. Thus, our use of the terms “transformation or phenotypic conversion” are constrained to observable changes in cell behavior linked to carcinogenesis *in vitro*, such as loss of cell density-dependent growth arrest, anchorage-independent growth and morphological changes associated with an epithelial to mesenchymal transition. ERK1-GFP protein expression was confirmed by Western blot (Figure 1, panel A bottom). The phosphorylation of both ERK1-GFP and endogenous ERKs was increased in response to growth factor treatment (10% FBS; 10 min), relative to respective quiescent controls (Figure 1, panel A top). ERK1-GFP translocated to the nuclear compartment following growth factor stimulation (Figure 1, panel B), consistent with nuclear translocation of ERK upon activation [16]. ERK is exported from the nucleus by the chromosome region maintenance protein 1 (CRM1) exporter [17] and treatment with a CRM1 inhibitor (20 nM LB; 30 min) resulted in nuclear retention of ERK1-GFP. Therefore, ERK1-GFP displays expected regulatory patterns in C10 cells.

**Figure 2:**
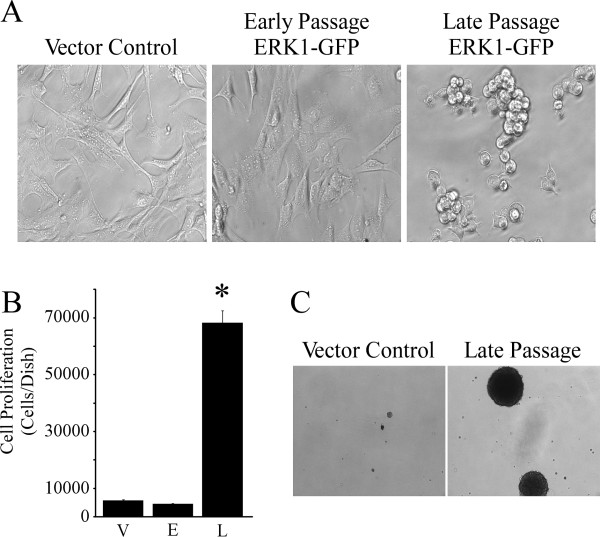
**Phenotypic conversion of C10 cells induced by ERK1-GFP.Panel A:** Late passage ERK1-GFP cells exhibit rounded morphology, relative to early passage ERK1-GFP cells and vector controls. **Panel B**: The proliferation of late passage ERK1-GFP cells (L) is not inhibited by cell-cell contact at confluence, while proliferation of early passage ERK1-GFP cells (E) and vector controls (V) are contact inhibited. *Significantly different from vector control. Values represent the mean ± se (n = 3). Similar results were observed in three independent experiments. **Panel C**: Late passage cells exhibit a qualitative change in their capacity to grow in soft agar, while vector controls show marginal anchorage-independent growth responses. Methods for the soft agar assay can be found in [20]. Similar results were observed in two independent experiments.

ERK1-GFP transduced cells exhibit a morphological change upon prolonged passaging (Figure 2A, compare early and late passage cells) which often accompanies cell transformation *in vitro *[18]. Total cell number was increased by approximately 11 fold in 5 day postconfluent cultures of late passage ERK1-GFP cells, relative to postconfluent vector control or early passage ERK1-GFP cells (Figure 2B), indicating loss of growth inhibition by cell-cell contact. Late passage ERK1-GFP cells grow in soft agar, while vector controls do not show significant anchorage-independent growth potential (Figure 2C), defined as previously described [19]. Collectively, late passage ERK1-GFP cells display multiple phenotypic alterations that suggest they have transformed to a malignant state.

**Figure 3:**
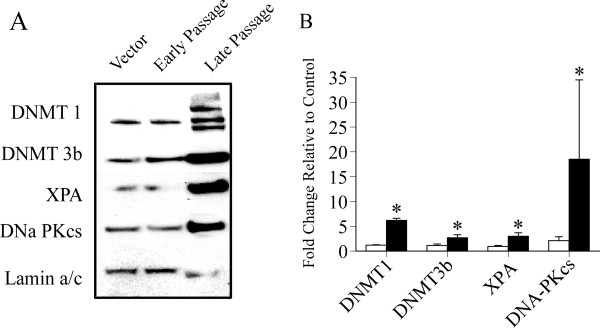
**Panel A: DNMT1, DNMT3b, XPA, and DNA-PKcs levels were increased in nuclear extracts prepared from late passage ERK1-GFP cells, relative to early passage ERK1-GFP cells and vector controls.** DNMT1 consistently exhibited multiple bands immunoreacting with anti-DNMT1 antibody in late passage ERK1-GFP cells. Lamin a/c was used as a loading control. **Panel B**: Graph illustrates pooled results from three independent experiments for early (white) and late (black) passage ERK1-GFP cells, relative to vector control (referenced as fold change value of 1). Values represent mean ± se (n = 3). *Significantly different from vector control. General methods for Western blot analysis can be found in [24].

ERK can regulate DNMT expression [21,22] which could impact epigenetic programming. Altered epigenetic programming is an attractive candidate in carcinogenesis because alterations in methylation of DNA are heritable and can lead to transcriptional dysregulation linked to neoplastic cellular changes [23]. Upon examination of DNMT expression patterns in our model, we observed a marked increase in DNMT1 and 3b isoforms in late passage ERK1-GFP cells, relative to early passage cells and vector controls (Figure 3A). DNMT3a was not detected by Western blot in our experiments (data not shown). DNMT1 was consistently characterized by the appearance of multiple bands immunoreacting with anti-DNMT1 antibody that were absent in early passage ERK1-GFP cells and vector controls. At present we do not know whether these bands represent alternative splice variants, degradation products, post-translational modifications or some combination. Thus, increased DNMT expression is latent in ERK transduced C10 cells (Figure 3A), suggesting that ERK is not directly regulating DNMT expression in this model, or that compensatory mechanisms prevent significant increases in DNMT expression patterns in early passage cells.

In previous studies we have used xeroderma pigmentosum complementation group A (XPA) as a loading control for nuclear extracts because the expression of this protein generally showed little change under a variety of experimental conditions. However, we observed a marked increase in the expression of XPA in late passage ERK1-GFP cells, relative to early passage cells and vector controls (Figure 3A). We subsequently defined the expression of DNA-dependent protein kinase catalytic subunit (DNA-PKcs) in late passage ERK1-GFP cells as an additional index for DNA damage signaling which was also increased (Figure 3A). Lamin a/c levels were not increased under these conditions and served as loading control. The combined results of three independent experiments are illustrated in Figure 3B. The reason for increased expression of DNA repair proteins is unclear. One possible interpretation may relate persistent ERK activation to genomic instability, which is a common feature of human cancers [25]. Genomic instability encompasses a broad array of chromosomal rearrangements and DNA damage events [26] that could generate signals leading to the regulation of repair proteins such as XPA and DNA-PKcs. ERK regulates NADPH oxidase activity [27], which is associated with a significant generation of oxygen free radicals [28] and chronic oxidative stress can induce genomic instability [29]. Alternatively, DNA-PKcs is hypothesized to play an important role in maintaining genomic stability [30] and the increase in DNA-PKcs may reflect effort to maintain stability in an unstable environment.

**Figure 4:**
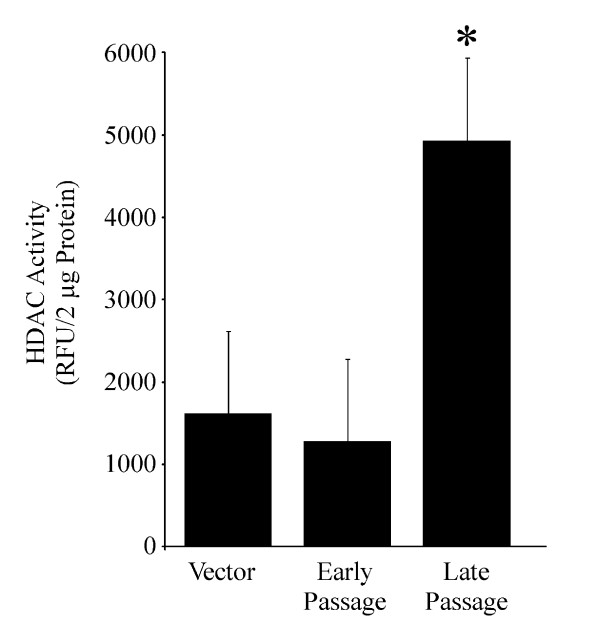
**HDAC activity in nuclear extracts from vector control, early and late passage ERK1-GFP cells.** Increased relative fluorescence units (RFU) is an index for increased HDAC activity, which was measured using the *Fluor de Lys – Green* kit (Enzo Life Sciences, Plymouth Meeting, PA) according to manufacturer’s directions. HDAC activity was increased in late passage ERK1-GFP cells, relative to early passage ERK1-GFP cells and vector control. ^*^Significantly different from vector control. Values represent the mean ± se (n = 3). Similar results were observed in three independent experiments.

DNMTs possess HDAC binding domains [31] and DNMT/HDAC systems are believed to be interdependent [32]. Therefore, we surveyed whether HDAC activity was altered under these conditions as an additional index for altered epigenetic programming that may be directly influenced by HDAC binding domains on DNMTs [31,32]. HDAC activity was significantly increased in late passage ERK1-GFP cells, relative to early passage cells and vector control (Figure 4). The abrupt increase in HDAC activity in late passage, but not early passage ERK1-GFP cells, is consistent with increased DNMT expression patterns and coupled HDAC regulation [31]. There is precedence for an ERK-DNMT-HDAC linkage in fear conditioning [33] and we hypothesize that these activities may also be aligned in carcinogenesis. Additional studies are required to dissect the relative amount of HDAC activity that is dependent on DNMTs in late passage cells.

**Figure 5:**
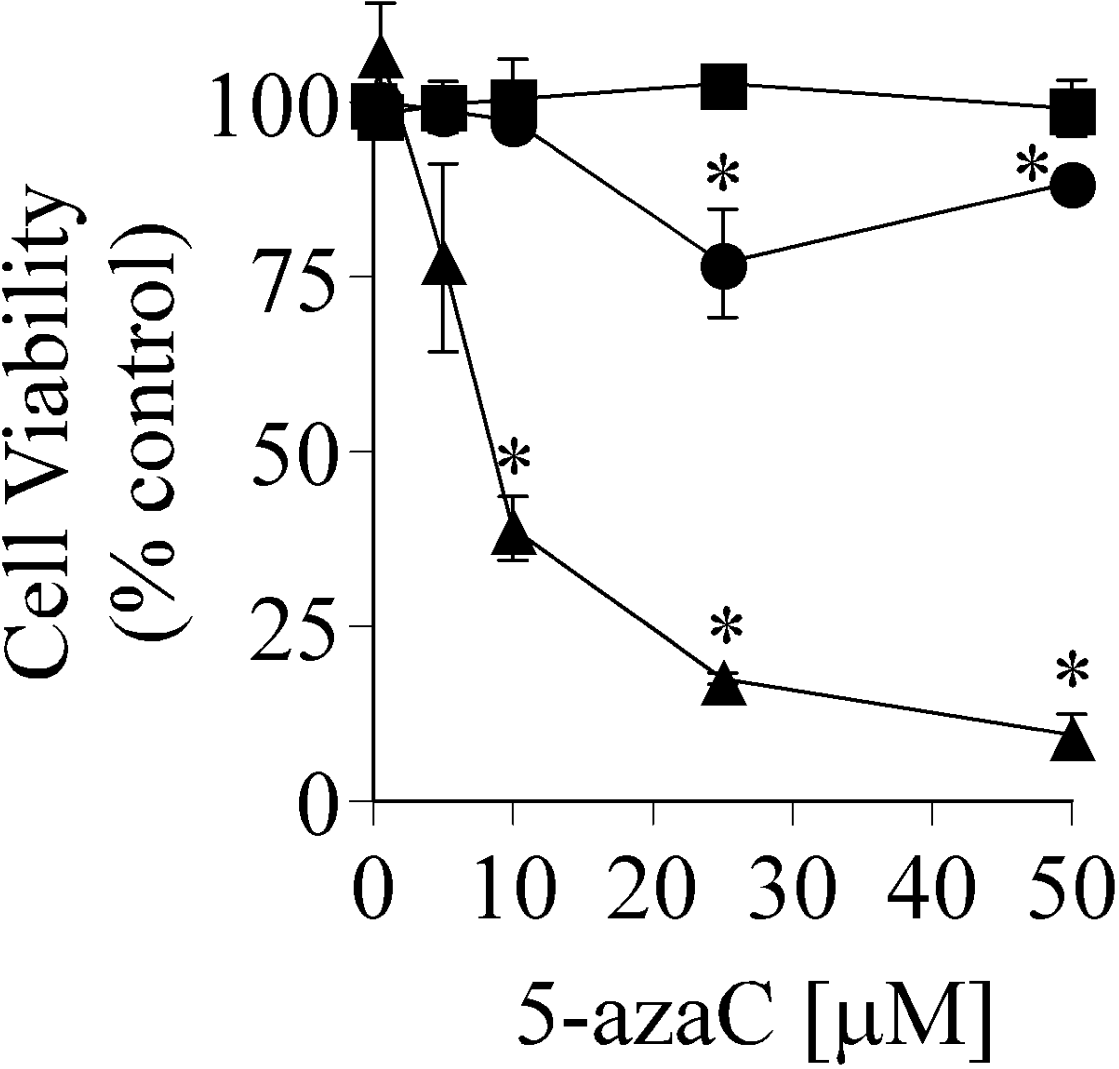
**5-azaC toxicity in ERK1-GFP transduced cells.** Vector control (square), early passage ERK1-GFP (circle) and late passage ERK1-GFP (triangle) were maintained in media supplemented with 5-azaC at the indicated concentrations for 7 days at which time cell viability was measured using a neutral red assay as described [34]. Values represent the mean ± se (n = 3). *Significantly different from vector control. Similar results were observed in three independent experiments.

To determine if increased DNMT expression was linked to cell fate regulation, we asked whether vector control, early and late passage ERK1-GFP cells were differentially sensitive to a DNMT inhibitor (5-azaC). Cells were treated with 0.5–50 μM 5-azaC for 7 days and cell viability was determined using a neutral red assay as previously described [34]. Cell viability was reduced in a dose-dependent manner by 5-azaC in late passage ERK1-GFP cells, but not in vector controls (Figure 5). Early passage ERK1-GFP cells displayed a small reduction in cell viability at the highest concentrations of 5-azaC (25–50 μM) employed. DNMT’s are under investigation as therapeutic targets for lung cancer [31]. Biomarkers that can predict when DNMT inhibitors may exhibit high efficacy could significantly aid in this effort. Because the C10 model developed here is sensitive to DNMT inhibitors, it may provide insight into molecular features that may serve as biomarkers, to the extent that such features are conserved in human cancers.

**Figure 6:**
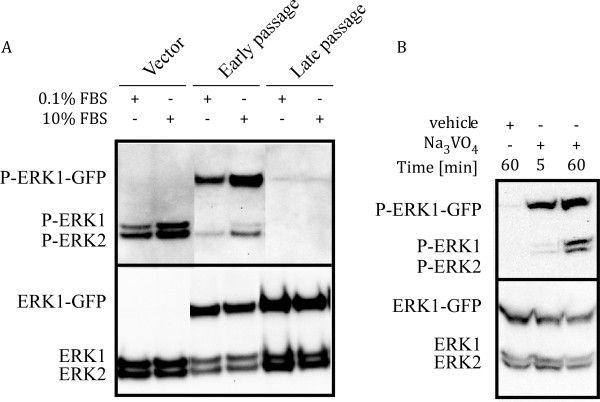
**Evidence for increased phosphatase activity in late passage ERK1-GFP cells.Panel A**: Western blot analysis of P-ERK levels in serum starved (0.1% FBS) vs 10% FBS stimulated cells. Treatment of cells with 10% FBS for 5 min results in increased P-ERK levels in vector control and early passage ERK1-GFP cells, as expected, while late passage ERK1-GFP cells display diminished P-ERK, but not total ERK levels. **Panel B**: Treatment of late passage ERK1-GFP cells with a broad spectrum phosphatase inhibitor (1 mM Na_3_VO_4_) for 5 or 60 min resulted in reappearance of P-ERK levels, suggesting that the decrease in P-ERK levels is due to increased phosphatase activity. Similar results were observed in two independent experiments

We consistently observed that late passage ERK1-GFP cells exhibited a marked reduction in phospho-ERK (P-ERK) levels, but not total ERK protein levels, relative to vector controls and early passage ERK1-GFP cells (Figure 6A). Treatment of serum starved cells (0.1% FBS) with 10% FBS for 5 min resulted in increased P-ERK levels in vector controls and early passage ERK1-GFP cells, which is the expected response to serum stimulation. Lack of P-ERK levels in late passage cells could result from either a general lack of signal transduction leading to ERK activation or an increase in phosphatase activity. We treated late passage ERK1-GFP cells with 1 mM sodium orthovanadate (Na_3_VO_4_) to determine whether a broad spectrum phosphatase inhibitor could restore P-ERK levels. P-ERK levels were restored within minutes of Na_3_VO_4_ treatment (Figure 6B), suggesting that the decrease in P-ERK levels associated with late passage ERK1-GFP cells was due to increased phosphatase activity.

## Conclusions

Ectopic expression of ERK alone is sufficient to induce phenotypic conversion of C10 cells and this model may provide insight into the underlying molecular determinants of this response. The window between early and late passage ERK-transduced variants that encompasses phenotypic conversion (approximately 15 passages) is a reasonable time frame to enable interrogative studies to define molecular determinants. Our expectation is that causal molecular processes will precede the appearance of the transformed phenotype and will be observed in early passage cells. At present, we have characterized changes in DNMT, DNA damage recognition and repair proteins and phosphatase activities that are selectively altered in late, but not early passage cells, suggesting they are secondary to transformation. Additional studies, perhaps with a more global screening approach, may provide insight into those molecular processes perturbed by ERK overexpression in early passage cells. Alternatively, because DNMTs are under investigation as therapeutic targets for lung cancer, the C10 model may provide insight into the molecular processes that confer sensitivity to DNMT inhibitors and regulate their aberrant expression.

## Abbreviations

5-azaC = 5-azacytidine; CRM1 = chromosome region maintenance protein 1; DNA-PKcs = DNA-dependent protein kinase catalytic subunit; DNMT = DNA methyl transferase; EMT = epithelial-mesenchymal transition; ERK = extracellular signal regulated kinase; ERK1-GFP = ERK1-green fluorescent protein; HDAC = histone deacetylase; LB = leptomycin B; MAPK = mitogen activated protein kinase; Na3VO4 = sodium orthovanadate; P-ERK = phospho-ERK; XPA = xeroderma pigmentosum complementation group A.

## Competing interests

The authors declare that they have no competing interests.

## Authors’ contributions

RS completed all experiments described under supervision by TW. All authors read and approved the final manuscript.
